# Changes in Content of Bioactive Compounds and Antioxidant Activity Induced in Needles of Different Half-Sib Families of Norway Spruce (*Picea abies* (L.) H. Karst) by Seed Treatment with Cold Plasma

**DOI:** 10.3390/antiox11081558

**Published:** 2022-08-11

**Authors:** Vaida Sirgedaitė-Šėžienė, Ieva Lučinskaitė, Vida Mildažienė, Anatolii Ivankov, Kazunori Koga, Masaharu Shiratani, Kristina Laužikė, Virgilijus Baliuckas

**Affiliations:** 1 Institute of Forestry, Lithuanian Research Centre for Agriculture and Forestry, Liepų Str. 1, Kaunas District, LT-53101 Girionys, Lithuania; 2 Faculty of Natural Sciences, Vytautas Magnus University, Vileikos Str. 8, LT-44404 Kaunas, Lithuania; 3 Center of Plasma Nano-interface Engineering, Kyushu University, Fukuoka 819-0395, Japan; 4 National Institutes of Natural Sciences, Center for Novel Science Initiatives, Tokyo 105-0001, Japan; 5 Institute of Horticulture, Lithuanian Research Centre for Agriculture and Forestry, Kauno 30, Kaunas District, LT-54333 Babtai, Lithuania

**Keywords:** antiradical assays, flavonoids, secondary metabolites, organic acids, food additives, health, natural extracts, low-temperature plasma, genetic selection, Norway spruce

## Abstract

In order to ensure sufficient food resources for a constantly growing human population, new technologies (e.g., cold plasma technologies) are being developed for increasing the germination and seedling growth without negative effects on the environment. *Pinaceae* species are considered a natural source of antioxidant compounds and are valued for their pharmaceutical and nutraceutical properties. In this study, the seeds of seven different Norway spruce half-sib families were processed for one or two minutes with cold plasma (CP) using dielectric barrier discharge (DBD) plasma equipment. At the end of the second vegetation season, the total flavonoid content (TFC), DPPH (2,2- diphenyl-1-picryl-hydrazyl-hydrate), and ABTS (2,2’-azino-bis (3-ethylbenzothiazoline-6-sulfonic acid)) antioxidant activity, and the amounts of six organic acids (folic, malic, citric, oxalic, succinic, and ascorbic) were determined in the needles of different half-sib families of Norway spruce seedlings. The results show that the TFC, antioxidant activity, and amounts of organic acids in the seedling needles depended on both the treatment duration and the genetic family. The strongest positive effect on the TFC was determined in the seedlings of the 477, 599, and 541 half-sib families after seed treatment with CP for 1 min (CP1). The TFC in these families increased from 118.06 mg g^−1^ to 312.6 mg g^−1^ compared to the control. Moreover, seed treatment with CP1 resulted in the strongest increase in the antioxidant activity of the needles of the 541 half-sib family seedlings; the antioxidant activity, determined by DPPH and ABTS tests, increased by 30 and 23%, respectively, compared to the control. The obtained results indicate that the CP effect on the amount of organic acids in the needles was dependent on the half-sib family. It was determined that treatment with CP1 increased the amount of five organic acids in the needles of the 541 half-sib family seedlings. The presented results show future possibilities for using cold plasma seed treatment in the food and pharmacy industries.

## 1. Introduction

In addition to its main chemical constituents (cellulose, hemicellulose, and lignin), wood contains a wide variety of low molecular mass compounds. In particular, wood compounds provide potential functionalities for pharmaceutical or nutritional products, cosmetics, beverages, wood adhesives, paints, wood protection agents, plant-protective products, and detergents [1]. 

Within conifers, the needles and new developing shoots of the *Pinacea* species are considered a rich natural source of secondary metabolites, such as phenols, flavonoids (e.g., kaempferol, quercetin, isorhamnetin, and myricetin), tannins, stilbenes, and terpenoids [2,3,4], as well as vitamins and minerals [4,5,6]. Woody plants synthesize a wide array of secondary metabolites [7,8,9,10], and many of them function as antioxidants. Since antioxidants neutralize free radicals or counteract their deteriorating effects [8,11], their use leads to beneficial effects on human health and maintaining wellness [12,13]. Many studies have shown that the *Pinacea* species is characterized by an abundance of compounds exhibiting valuable pharmaceutical and nutraceutical properties [14,15]. Norway spruce (*Picea abies* (L.) H. Karst) needles are a high source of vitamin C and minerals [2,4,6,16], carotenoid, and chlorophylls [17,18]. Spruce needles have long been used by indigenous tribes for relieving coughs and sore throats [9]. The concentrations of the isolated bioactive compounds detected in Norway spruce tissues are dependent on numerous factors, such as the wood age and growing period, the time of harvesting, and the genetic and geographical background [6].

The applications of a new field of science—plasma and magnetic biology—due to its unique potential, have expanded into different research areas. Plasma treatment can stimulate biological processes in various organisms, further acting as a sterilizing agent. These can be key aspects for developing and using plasma technologies in the food industry, medicine, ecology, and agriculture [19]. Research on cold plasma (CP) applications for agriculture is relatively new but an extremely rapidly developing area, as reflected by the large number of recent reviews [20,21,22,23,24,25]. It has been demonstrated that seed treatments with CP may have significant positive effects on germination, plant biochemical processes, photosynthesis [26,27], and biomass growth [27,28] and could increase harvest yields of numerous plant species. However, although certain important molecular details have recently been revealed, the detailed mechanisms of these effects have not yet been elucidated [21,22,29]. Moreover, pre-sowing seed treatment with physical stressors induces changes in the germination and plant growth, further increasing the concentration of biologically active compounds. Substantial increases in the production of secondary metabolites in seedlings growing from CP-treated seeds have been reported [28,30], as well as increases in chlorophyll contents [26,31,32] and antioxidant activity [33].

Studies have shown that the ability of different half-sib families of woody plants to synthesize secondary metabolites and accumulate them are diverse, and therefore, genetic selection is appropriate [34,35,36]. Thus, when natural seed mixtures (composed of seeds from different half-sib families) are investigated, different individual responses are averaged and can even eliminate each other; therefore, the possibility of estimating the genotype-dependent effects is very limited. 

Our earlier study showed that the treatment of Norway spruce seeds with atmospheric dielectric barrier discharge (DBD) plasma for 1 or 2 min induced genotype-dependent changes to the height, the content of total phenolics, and the photosynthetic pigments in young seedlings (in the second vegetation season) of different Norway spruce half-sib families [37]. For example, in only some of the seven families (457, 477, and 577), seed treatment with cold plasma for 2 min (CP2) had a significant positive effect on the height of the seedlings, but the heights of the seedlings were not affected in the other families (463, 541, 548, and 599). In addition, seed treatment with DBD plasma for 1 min (CP1) had a significant positive effect on the total phenolic content (TPC) in only the 477, 463, and 541 half-sib families of Norway spruce (increasing from 11 to 14% in comparison to the control). Moreover, it was established that seed treatment with CP2 strongly increased (50–70% compared to the control) concentrations of chlorophylls and carotenoids in the needles of the 477 half-sib family seedlings; however, the effects on the pigment content were absent in the other half-sib families. 

The results of a detailed study on differences in the bioactive compounds (including the total flavonoid content and six organic acids) and antioxidative activity in Norway spruce needles from seven half-sib families grown from control seeds were recently reported, and substantial variation dependent on the half-sib family was revealed [38]. In this study, we aimed to examine what changes in the antioxidative capacity and content of metabolites in Norway spruce needles were induced by seed treatment with DBD plasma (CP1 and CP2) in seven half-sib families of Norway spruce. Such a study is important for gaining more information on possible plasma applications leading to improved forestry production, both for increased seedling establishment or growth (due to enhanced stress resistance) and to the enhanced production of the pharmacologically valuable compounds exerting a beneficial impact on human health. Unfortunately, the existing knowledge on changes induced in the phytochemical machinery of plants by seed treatment with cold plasma is still very scarce. In order to fill this knowledge gap, an experiment was designed to reveal the implications and interactions among physical stressors, half-sib families, and the concentrations of valuable bio-based compounds in Norway spruce needles. 

## 2. Materials and Methods

### 2.1. Samples Collection

The collection of seeds from Norway spruce half-sib families, the method used for seed treatment with cold plasma, and the conditions for seedling cultivation were described in detail in an earlier study [37]. Norway spruce seeds from 7 different half-sib families were collected from the Trakai regional division’s second-generation spruce seed orchard. The collected seeds were stored in a refrigerator (+4 °C) until the experiment. 

### 2.2. Seeds Treatment with Cold Plasma 

The seeds of each Norway spruce half-sib family were exposed to cold plasma or low-temperature atmospheric dielectric barrier discharge (DBD) plasma (duration of exposure of 1 or 2 min), as described by Sirgedaitė-Šėžienė, 2021 [37]. 

The seeds were treated with CP using a controlled DBD discharge device produced at Kyushu University, Japan [39], with a homogeneous treatment area of 4 × 4.38 cm^2^. The discharge voltage, current, and power density were 7.0 kV, 0.2 A, and 3.1 W/cm^2^, respectively. The treatment times were 1 and 2 min at a distance of 5 mm between the seed surface and the electrode plate. The seeds were treated at atmospheric pressure, room temperature, and 45–55% air humidity.

### 2.3. Cultivation of Seedlings and Sample Collection for Experiments

After the seed treatment with CP, the seeds were left for 4 days at room temperature. For each experimental group, 80 seeds were treated on 4 June 2018. A total of 80 untreated seeds from each half-sib family were used as the control. The total amount of seeds used for this experiment was 1680 (7 genotypes × 3 groups × 80 seeds). The Norway spruce seeds were sown in cassettes filled with peat substrate and cultivated in blocked randomized 40-cell cassettes. They were grown in greenhouse conditions for the first two months, and then placed outdoors in an open light area. A nursery with four tunnel-type greenhouses with dimensions of 16 × 80 m (1280 m^2^) and controlled environment conditions (temperature during the summer: 25–32 °C during the day and not lower than 10 °C at night) was used. Samples of the seedling needles were collected by taking 3–5 needles from each of the 40 seedlings in each group, as described earlier [37]. The total amounts of the samples (500 mg) were randomly divided into three equal parts according to weight in order to form one or three biological replicates from the control and each experimental group at the end of the second vegetation season (Table 1). Biochemical analyses of the total flavonoid content, DPPH and ABTS scavenging activity, and organic acids were carried out to evaluate the biochemical compositions and antioxidant activities. 

### 2.4. Sample Preparation

Amounts of 500 mg of fresh needles samples were homogenized using an analytical mill (Laboratory Equipment, Staufen, Germany), mixed with 10 mL of 75% MeOH (methanol) and shaken at room temperature for 24 h using a Kuhner Shaker X electronic shaker (Adolf Kühner AG, Birsfelden, Switzerland). After 24 h, the needle extracts were filtered using Rotilabo ^®^-113A (Ø 90 mm) filter papers (Carl Roth, Karlsruhe, Germany). 

### 2.5. Quantification of Total Flavonoid Content

The total flavonoid content (TFC) was determined using the spectrophotometrical method based on the formation of flavonoid complexes with aluminum ions Al (III) [38]. An extract solution (1 mL) was mixed with 0.3 mL of 5% (*w*/*v*) NaNO_2_, and after 5 min, 0.5 mL of the AlCl_3_ (2% *w*/*v*) was added. The obtained sample was mixed, and after 6 min, it was neutralized with 0.5 mL of 1 M NaOH solution. The absorbance was measured at 470 nm using the Synergy HT Multi-Mode Microplate Reader (BioTek Instruments, Inc., Bad Friedrichshall, Germany). The flavonoid content was expressed in milligrams of catechin per gram of fresh material weight (mg CE/g). The standard calibration curve equation for samples was y = 11.616x + 0.0634 (*R*^2^ = 0.9983). 

### 2.6. Detection of Radical Scavenging Activity Using ABTS and DPPH Assays 

An antiradical assay was carried out with the spectrophotometrical method using the radical cation ABTS 98% (2,2′-azino-di-[3-ethylbenzthiazoline sulphonate]), generated following [40]. The ABTS (0.056 g) was dissolved in 50 mL of dH_2_O. ABTS radical cation was produced by reacting ABTS stock solution with 200 µL of 70 nM K_2_S_2_O_8_ (0.1982 g K_2_S_2_O_8_ dissolved in 10 mL dH_2_O). The mixture was held in a dark room at room temperature for 16 h before it was used. After 16 h, the mixture was diluted with dH_2_O until it reached 0.700 ± 0.2 absorbance (734 nm). All measurements were performed at room temperature. The sample (50 µL) was mixed with 2 mL of the ABTS solution and placed in the dark. After 10 min, the absorbance was measured using a Synergy HT Multi-Mode Microplate Reader (BioTek Instruments, Inc, Germany). Trolox was used as the standard. The standard calibration curve equation was y = 0.2734x + 0.0304 (*R*^2^ = 0.9842). The radical scavenging activity was calculated from the Trolox equivalent per gram of fresh material, using Equation (1).

(1) $$\mathrm{TE}=\frac{c\times V}{m}$$

where *c* = the Trolox concentration (mM/mL); *V* = the extract volume (mL); *m* = the fresh material amount (g).

The free radical scavenging capacity in the methanol extracts of the needle samples was also spectrophotometrically determined by DPPH > 97% (2,2–diphenyl–1–picrylhydrazyl) assay, as described in [41]. The DPPH (7.9 mg) was dissolved in 200 mL of methanol (100%). Then, 100 μL samples were mixed with 1 mL of DPPH and 400 μL of MeOH (75%). After 16 min in the dark, the absorbance was measured at 515 nm using a Synergy HT Multi-Mode Microplate Reader (BioTek Instruments, Inc, Germany) against an equal amount of DPPH and methanol as a blank. The standard calibration curve equation was y = 0.2074x − 0.004 (*R*^2^ = 0.9907). The radical scavenging activity was calculated as the antiradical Trolox equivalents per gram of fresh material, using Equation (1). 

### 2.7. Quantification of Organic Acids by HPLC

The organic acid contents were analyzed by HPLC following the method of Wang et al. (2014) [42]. The Shimadzu 10A (Japan) system was used in the reverse phase with the refraction index detection at a wavelength of 210 nm. Approximately 0.5 g of the sample was homogenized in distilled water (1:10 *w*/*v*). The solutions were condensed in a heated water bath for 30 min at 50 °C. The extract was clarified by centrifugation at 10,000 rpm for 15 min. Then, the solution was filtered through a 0.22 µm PTPE syringe filter (VWR International, Radnor, PA, USA). The sample separation was performed using a Lichrosorb RP-184.6 × 250 mm and a 5 µm column (Altech). A volume of 10 µm of the sample was injected into the column at a temperature of 25 °C. The isocratic elution method was used. A mobile phase composition was tested with 0.05 M of sulfuric acid at a flow rate of 0.5 mL min^−1^. The analysis time was 20 min. The calibration method (*R*^2^ < 0.99) was used for each organic acid quantification (mg g^−1^ in FW). The standard calibration curve equations were as follows: ascorbic acid: y = ax^2^ + bx + c (where a = 1.58276 × 10^9^; b = 1.91555 × 10^8^; c = 248,409 (*R*^2^ = 0.9988971)); oxalic acid: y = ax^2^ + bx + c (where a = −3.06102 × 10^9^; b = 5.52036 × 108; c = −210236 (*R*^2^ = 0.9999016)); folic acid: y = ax^2^ + bx + c (where a = 1.07988 × 10^8^; b = 6.21486 × 10^7^; c = −19479.1 (*R*^2^ = 0.9989302)); malic acid: y = ax^2^ + bx + c (where a = −8.53699 × 10^8^; b = 7.07799 × 10^8^; c = −57425.2 (*R*^2^ = 0.9999703)); citric acid: y = ax^2^ + bx + c (where a = 1.61593 × 10^9^; b = 6.92654 × 10^7^; c = 227,696 (*R*^2^ = 0.9958250)); succinic acid: y = ax^2^ + bx + c (where a = 1.61593 × 10^9^; b = 6.92654 × 10^7^; c = 227,696 (*R*^2^ = 0.9958250)).

### 2.8. Statistical Data Analysis

SAS software (Version 9.4, Cary, NC, USA, 2012) was used for the statistical analysis. All analyzed traits followed a normal distribution. Variance components of the traits were calculated by applying the SAS MIXED procedure (REML method). Model 2 was applied for the analyses of the treatment and family effects:

*Y*_*ijk*_ = *µ* + *S*_i_ + *F*_*j*_ + *E*_*ijk*_ (2)

where *µ* is the grand mean, *S_i_* is the random effect of treatment *i*, *F_j_* is the random effect of family *j*, and *E_ijk_* is the residual error.

Model 3 includes GxE interaction:

*Y*_*ijk*_ = *µ* + *S*_*i*_ + *F*_*j*_ + *SF*_*ij*_ + *E*_*ijk*_ (3)

where *µ* is the grand mean, *S_i_* is the fixed effect of treatment *i*, *F_j_* is the random effect of family *j*, *SF_ij_* is the random effect of treatment *i* and family *j* interaction, and *E_ijk_* is the residual error.

The TTEST procedure (*t*-test) was used for pairwise comparisons of the treatments among the experimental groups CP1, CP2, and the control (* *p* < 0.05; ** *p* < 0.01; *** *p* < 0.001). The same method was applied for the comparison of each family trait with the control as well as for the comparison between families. The PC-ORD5 software was used in conducting principal component analysis (PCA, Pearson type). The coordinates for the biplot represent the tips of line segments radiating from the centroid. 

## 3. Results

**Figure 1:**
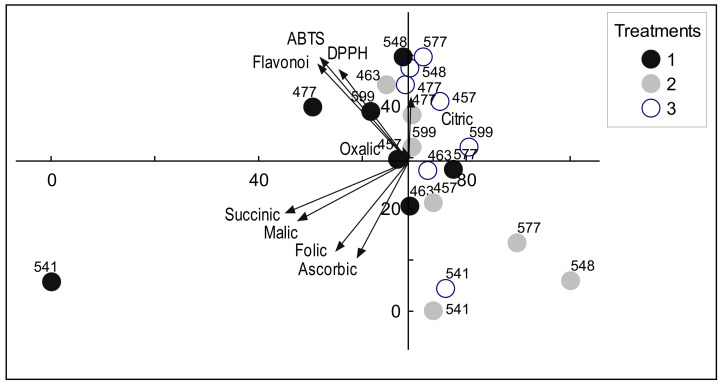
PCA biplot of the first and second components for the antioxidant compounds and amount of organic acids (on half-sib family mean level). Treatments: 1—seed treatment with cold plasma 2 min (CP2); 2—seed treatment with cold plasma 1 min (CP1); 3—control group. The family number is shown next to the circle.

The results of the principal component analysis (PCA) are presented in Figure 1. Two components explained 48.98% of the total variation of the dataset. The flavonoids, DPPH, and ABTS are grouped together. 

Flavonoids and ABTS have the highest factor loading estimates and are presented in Table 2.

### 3.1. Total Flavonoid Content

**Figure 2:**
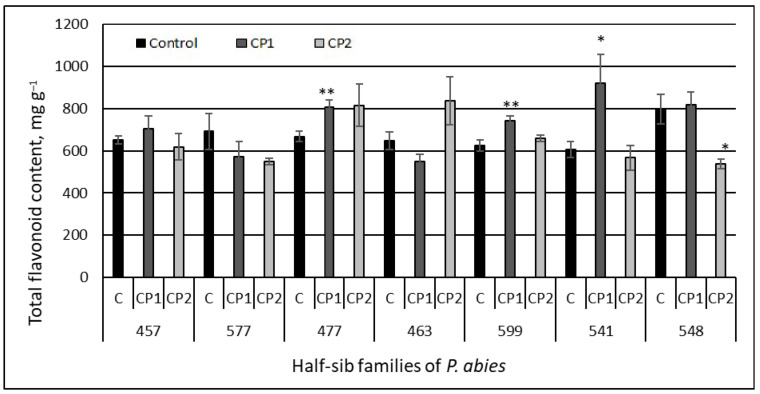
Total flavonoid content in the needle extracts of Norway spruce seedlings growing from control and treated seeds. C – control, CP1 – seeds treated with cold plasma 1 min., CP2 – seeds treated with cold plasma 2 min. The average ± SE (*n* = 63). The asterisks indicate the statistical significance of the difference between the treated group and the control in each half-sib family (* *p* < 0.05; ** *p* < 0.01).

The TFC analysis did not reveal statistically significant differences in the flavonoid contents between the control groups of Norway spruce half-sib families (Figure 2), except for a higher TFC (by approximately 23%) in the 548 family as compared to 541 and 599. The amount of TFC in the needles of the control Norway spruce seedlings varied from 606.7 ± 36.77 (in the 541 family) to 797.64 ± 69.98 (in the 548 family). 

However, the seed treatment with plasma induced different TFC changes in the needles of the half-sib families. There was no statistically significant difference in the TFC between the control and CP-treated groups in the seedlings from the three families (457, 577, and 463), but the CP1 (but not CP2) treatment increased the TFC in the needles in the seedlings from the 477, 599, and 541 families (by 17, 16, and 34% compared to the control, respectively). The only negative effect of the CP treatment on the TFC was observed in the 548 family seedlings growing from CP2-treated seeds. This family was characterized by the highest TFC in the control, but the TFC in the CP2 group seedlings was substantially reduced (by 33%) (Figure 2). The half-sib family variance component for the TFC concentration was 11.9%, while the treatment variance component was 2.6% (Model 2).

### 3.2. Antiradical Activity

**Figure 3:**
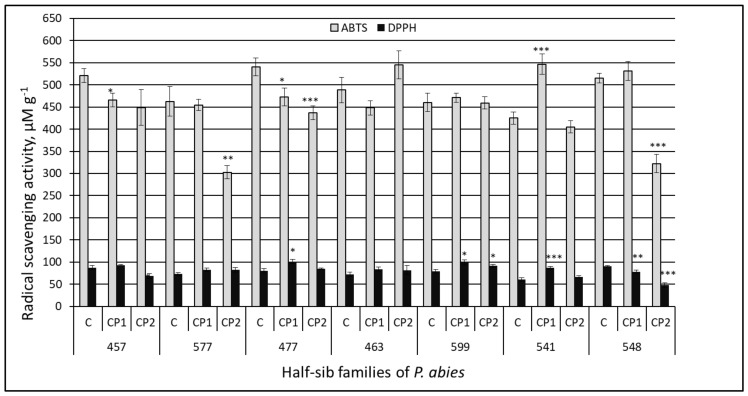
Antioxidant activity in the needle extracts of Norway spruce seedlings growing from control and treated seeds detected by DPPH and ABTS methods. C—control; CP1—seeds treated with cold plasma 1 min; CP2—seeds treated with cold plasma 2 min. The average ± SE (*n* = 63). The asterisks indicate the statistical significance of the difference between the treated group and the control in each half-sib family (* *p* < 0.05; ** *p* < 0.01; *** *p* < 0.001).

The antiradical activity in the extracts of the Norway spruce needles was determined by two different methods—DPPH and ABTS tests (Figure 3).

In comparison with the ABTS test, the DPPH binding revealed more differences in the antioxidative capacity between the control groups of the Norway spruce seedlings from different half-sib families. For example, according to the DPPH test, the seedlings from the 541 family had statistically lower antioxidant activity compared to the seedlings from the 457, 577, 477, and 599 families (by 31, 17, 24, and 23%, respectively), whereas the ABTS test indicated only differences between the 541 and two other (457, 477) families (the 541 antioxidant activity was lower by 18–21%). Similarly, according to the DPPH binding, the activity was higher in the seedlings from the 548 family compared to the seedlings from the 577, 463, 599, and 541 families (by 19, 21, 13, and 33%, respectively), but the ABTS test showed that the antioxidative capacity of the needles in the 548 line was higher compared to the 599 and 541 half-sib families (by 11 and 18%, respectively) only. According to the DPPH (but not the ABTS) test, the antioxidative activity in the needles of the 457 family seedlings was 18% higher compared to that in the 463 seedlings. On the other hand, only the ABTS test revealed that the antioxidative capacity in the needles of the 599 family was statistically lower (12–15%) in comparison to the activity detected in the 457 and 477 families. 

In all the Norway spruce families, the CP-induced changes depended on both the used method and the treatment dose. In addition, the 541 family had statistically (*p <* 0.001) higher radical scavenging activity after the CP1 treatment using both methods: ABTS (increase of 23%) and DPPH (increase of 30%). The DPPH test revealed similar positive effects of the seed treatment with CP1 on the antioxidative activity in the seedlings from the 477 (increase of 21%), 599 (increase of 21%), and 541 (increase of 30%) families; however, the only positive effect revealed by the ABTS test was in the 541 family (22%). The only negative CP1-induced effect based on the DPPH test was observed in the 548 family extracts (14%), while the ABTS test indicated small negative effects (11–13%) in the seedlings from the 457 and 477 half-sib families of Norway spruce.

The only positive effect of CP2, compared to the control, was revealed by the DPPH test in the 599 half-sib family (14%). The antioxidative activity remained unchanged in the CP2-treated groups from the 577, 477, 463, and 541 families (according to the DPPH binding), or from the 457, 463, 599, and 541 families (according to the ABTS test). The seed treatment with CP2 caused negative effects in more families, and the effects were stronger compared to the CP1 effects: the DPPH binding decreased in the seedlings from the 457 and 548 families (by 21 and 47%, respectively); the ABTS test indicated decreased antioxidative activity in the 577, 477, and 548 families (by 35, 19, and 37%, respectively). 

The analysis of the variance components of the treatment effect showed that the strongest effect was on the antioxidative activity detected by the ABTS test and the weakest on the flavonoid concentration. The statistical analysis revealed that the family effect was strong for the flavonoid concentration and antioxidative activity determined by the DPPH binding test. The results of the application of the statistical Model 2 reveal the magnitude of the interaction between the treatment and the family. The interaction effect exceeded the family by 4–10-fold depending on the trait. The strongest effect was in the case of the ABTS test results, which reached 43.0% of the total variance.

### 3.3. Amount of Organic Acids

**Figure 4:**
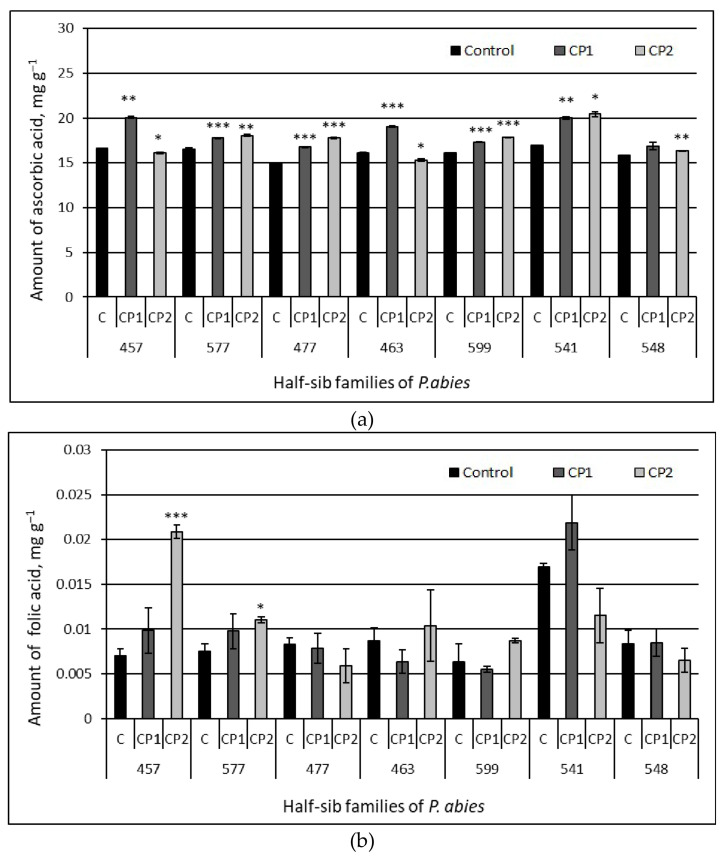

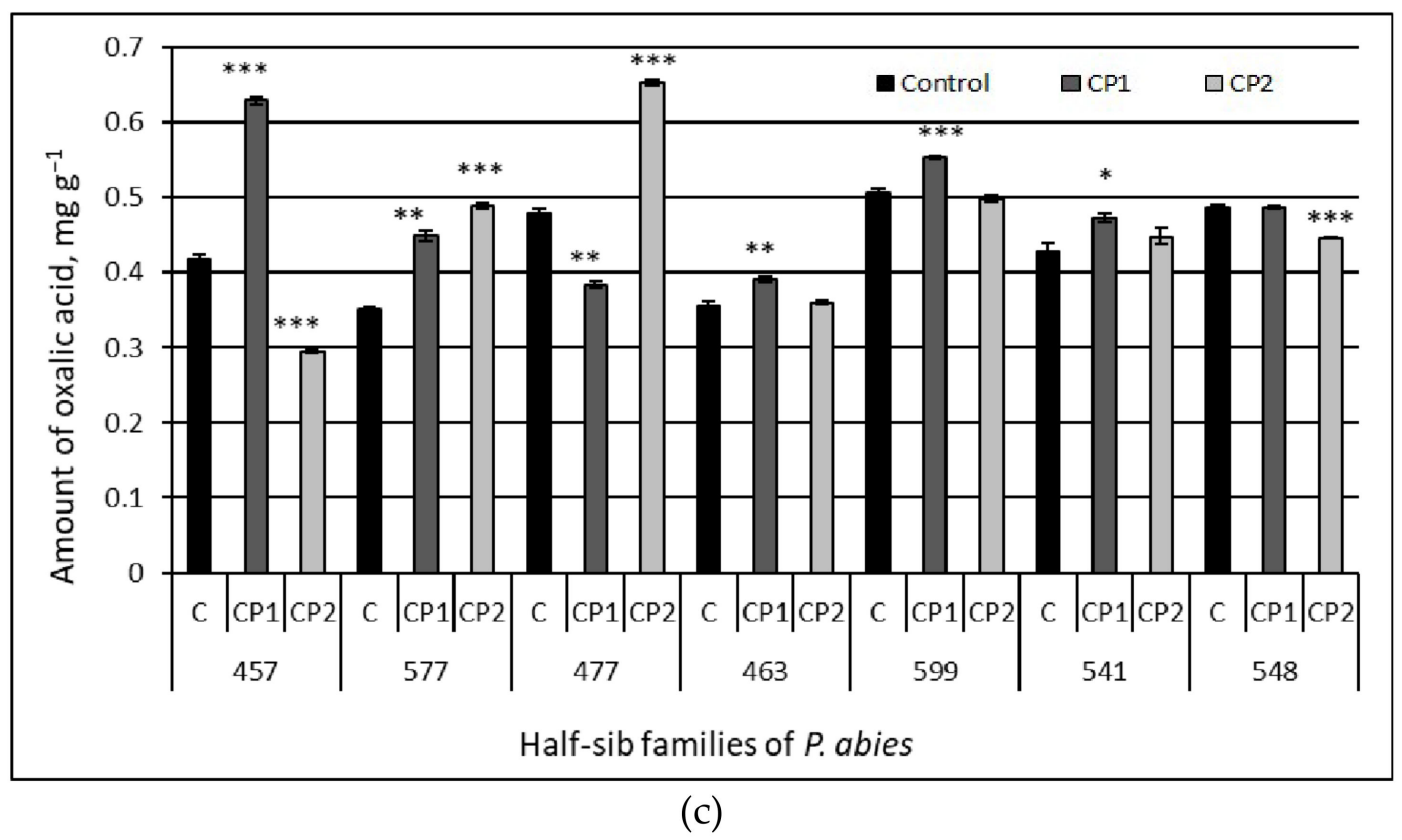
Amount of organic acids – ascorbic acid (**a**), folic acid (**b**) and oxalic acid (**c**) in the needle extracts of Norway spruce seedlings growing from control and treated seeds. C – control, CP1 – seed treatment with cold plasma 1 min., CP2 – seed treatment with cold plasma 2 min. The average ± SE (*n* = 21). The asterisks indicate the statistical significance of the difference between the treated group and the control in each half-sib family (* *p* < 0.05; ** *p* < 0.01; *** *p* < 0.001).

**Figure 5:**
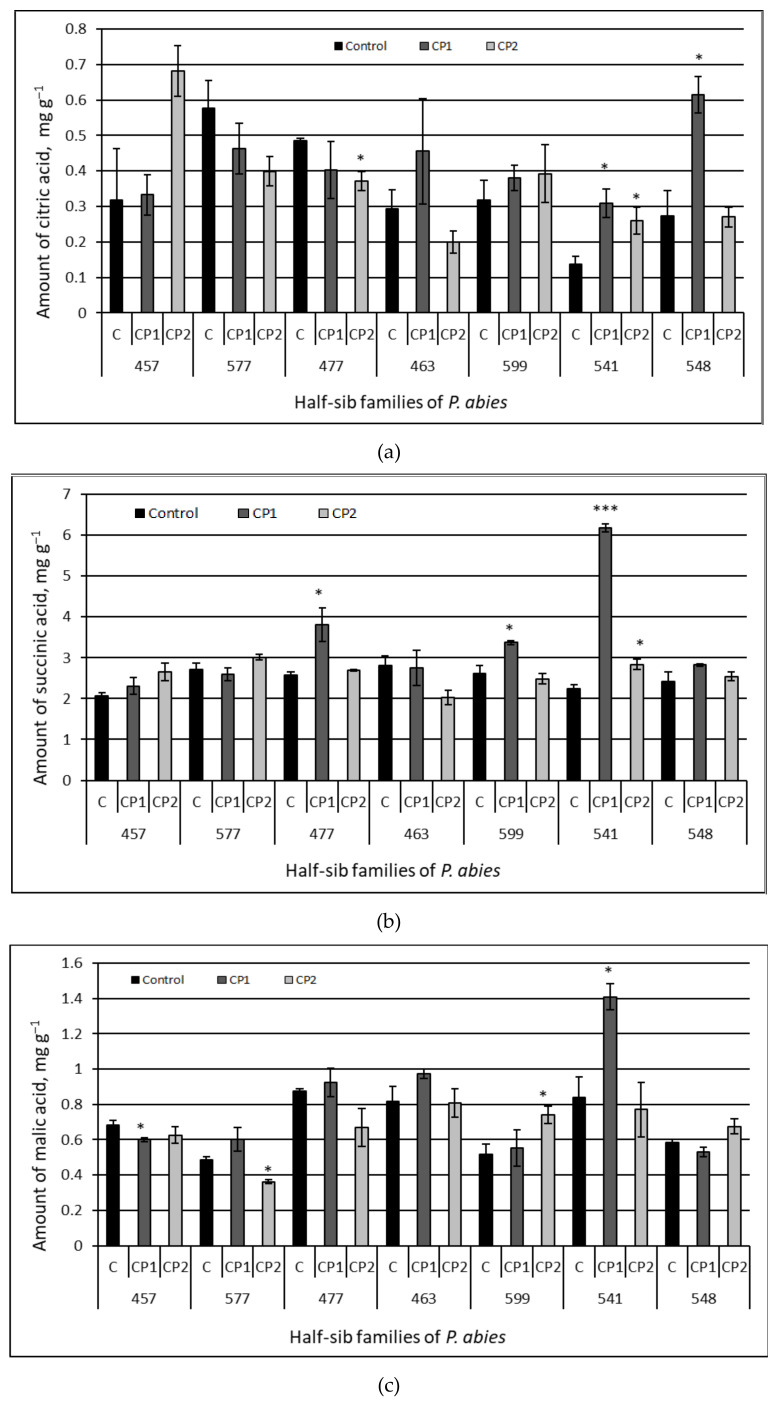
Amount of organic acids – citric acid (**a**), succinic acid (**b**) and malic acid (**c**) in the needle extracts of Norway spruce seedlings growing from control and treated seeds. C – control, CP1 – seeds treated with cold plasma 1 min., CP2 – seeds treated with cold plasma 2 min. The average ± SE (*n* = 21). The asterisks indicate the statistical significance of the difference between the treated group and the control in each half-sib family (* *p <* 0.05; *** *p* < 0.001).

The contents of six organic acids in the needles of the seedlings from the different half-sib families of Norway spruce grown from the control and CP-treated seeds were determined by HPLC analysis. The results are presented in Figure 4 ((a) ascorbic; (b) folic; and (c) oxalic acids) and Figure 5 ((a) citric; (b) succinic; and (c) malic acids), and the pairwise comparison data are provided in Table 3. 

The obtained results show that the content of ascorbic acid in the needles of the control seedlings varied in a range between 14.95 ± 0.03 mg g^−1^ (477 family) and 16.92 ± 0.01 mg g^−1^ (541 family) and was the largest in comparison to the contents of all other organic acids (Figure 4 and Figure 5). Although the extent of variation in the content of ascorbic acid was small, differences in its contents among the majority of the half-sib families were statistically significant, as was the interaction between the contents of organic acids in the control and families. The abundance of other organic acids decreased in the order of succinic > malic > oxalic > citric > folic acid. The largest extent of variation in the amounts of the six organic acids in the control seedlings among the Norway spruce half-sib families was observed in the contents of the folic (Figure 4b), citric (Figure 5a), and malic (c) acids. The citric acid amounts in the control extracts were 76% higher in the 577 family compared to the 541 family (Figure 5a). The control needles from the 477 family seedlings contained about 22 and 45% more malic acid in comparison to the needles from the 457 and 577 families, respectively. The content of folic acid in the needles of the 541 family seedlings was much higher compared to all other families (except 599 family) (e.g., 59% exceeded that in the 457 family). 

The effects of the seed treatment with cold plasma on the contents of the six organic acids also varied among the half-sib families. The CP1 treatment induced positive effects on the amount of ascorbate in the six families (in the 548 family only, such an effect was not statistically significant). The extent of the positive effect varied between 7% (in the 577 and 599 families) and 17% (in the 457 family) (Figure 4a). Increases in the ascorbate content (from 6 to 17%) were induced by the CP2 treatment in the needles from the 577, 477, 599, 541, and 548 families and slight decreases (4–5%) were detected in the 457 and 463 families, respectively. The amount of folic acid in the needles from the 477, 463, 599, 541, and 548 families was not affected by the seed treatment with CP (Figure 4b). The only effect of the seed treatment with CP1 was observed in the 541 family seedlings, where the content of folic acid increased by 23% compared to the control; however, the CP2 treatment induced a decrease in the folic acid content by 32% in the needles from the same family. Strong positive effects of the CP2 treatment on the folic acid content were observed in the needles from the 457 (3-fold increase) and 577 (32%) families. Seed treatments induced changes in the contents of oxalate in the needles in all seven half-sib families (Figure 4c). The CP1 treatment increased the oxalate content in the 457, 577, 463, 599, and 541 families (by 33, 22, 9, 8, and 10%, respectively) but decreased it in the 477 family (20%). Positive effects of CP2 on the oxalic amount were observed in the 577 and 477 families (28 and 27%, respectively), but in the needles from the 457 and 548 families, the CP2 decreased the oxalic amount (30 and 8%, respectively). 

The effects of the treatments on the amount of intermediates in the tricarboxylic acid cycle (citric, succinic, and malic acids) in the needles were also different in the seven half-sib families of Norway spruce (Figure 5). The citric acid content in the needles from the seedlings of the 457, 577, 463, and 599 families remained unchanged by the CP treatments, but underwent strong changes in other families: a 2.2–2.3-fold increase in the citric acid content was detected in the CP1 treated-groups of the 541 and 548 families; and a 1.9-fold increase occurred in the CP2-treated groups of the 541 family (Figure 5a). The only negative effect on the citric acid content was observed in the CP2-treated group of the 477 half-sib family (23%). The strongest positive effects of the treatments on the content of the succinic acid were observed in the 541 family: CP1 induced an increase of 64%, and CP2 induced an increase of 21%. Positive effects on the succinic acid amount were also detected in the CP1-treated groups of the 477 and 599 families (by 32 and 22%, respectively). In contrast, the seed treatment with CP2 did not change the content of succinic acid in comparison to the control in the other six half-sib families, and neither did CP1 in the needles from the CP1-treated groups of the 457, 577, 463, and 548 families (Figure 4b). The treatments did not change the content of malic acid in the needles from the 477, 463, and 548 families (Figure 5c) and had different effects on other families: CP1 increased the amount of malic acid in the 541 family (40%) but decreased it in the 457 family (12%); the CP2 treatment caused an increase in the 599 family (30%) and a decrease in the 577 family (26%) needles.

The evaluation of the components of variation according to the second statistical model revealed that the ascorbic, folic, and oxalic acids had similar and high values of the family effect (24.0–30.4%). Among all the studied organic acids, the family effect was the strongest for the malic acid content. The family component of variation for the citric and succinic acids was less than 20%. The treatment effect exceeded the family effect in only two cases, namely the ascorbic and succinic acids. However, looking at the results of the third statistical model, the largest variance component of the G × E interaction was for oxalic acid, followed by succinic and ascorbic acids (above 50%). By definition, the interaction becomes apparent when different genotypes respond differently to changes in the environment (in our case, treatment). Only in the case of malic acid did the family component exceed the G × E interaction component (Model 3).

## 4. Discussion

The production of the number of secondary compounds, which are biosynthesized from primary metabolites and accumulated in plant cells, could be induced with biotic, abiotic elicitors, or signaling molecules [43,44,45,46]. For many years, humankind has used physiological adjustments in plants to improve the biosynthesis of biologically active compounds, which are useful in the production of drugs, supplements, or for direct use of the plants as herbal medicines. Approximately 25% of the drugs human beings use are produced from medicinal plants [47,48]. Among woody plant species, Norway spruce is known as one of the richest in its diversity of chemical compounds [2,4,38,49]. Our previous research showed that seed treatment with cold plasma (CP), as an innovative technology, can induce the accumulation of phenolic compounds and photosynthetic pigments in the needles of Norway spruce, and that the extent of such an effect depends on half-sib family properties [37]. Such bioactive compounds could be used for their nutritional value, as food additives, and as biochemicals for industrial use [50,51,52,53]. Based on recent publications, changes in free radicals are related to the phenolic background in plants, and are one of the essential parameters of different kinds of human diseases [54,55], have preventive properties against cancer and heart disorders [56], and improve the antiradical level in blood plasma [57]. Our research noted that plant genetic selection is a relevant background for identifying plant characteristics and adjusting them in the production of supplements. The findings of other authors [36,58,59,60] indicate that the variability of the secondary metabolites is not only exclusively related to the different geographical or morphological differences, but also to the genotype of the plants. 

Moreover, the present study revealed that the seed treatment with CP also induced changes in the concentration of other valuable bio-based compounds, such as flavonoids and organic acids, as well as the antioxidant activity in the needles. The analysis of the variance components performed in this study showed that the family effect was strong for the flavonoid concentration. The short-term treatment with CP had a statistically significant positive effect on the flavonoid (TFC) increase in the needles in the seedlings from the 477, 599, and 541 half-sib families compared to the other Norway spruce half-sib families. The antioxidant capacity increased with an increase in TFC content. This might be related to the flavonoid’s ability to markedly enhance antioxidant properties. In addition, the Pearson correlation coefficient between the total flavonoid content and the antioxidant activity (ABTS test) induced by the seed treatment with CP2 shows a significantly positive correlation (r = 0.458; *p* < 0.05). Moreover, other authors have also indicated that antioxidant activity is directly related to the concentration of secondary compounds in certain *Pinaceae* species, e.g., *Pinus mugo* [61] and *Picea abies* [6]. Speisky et al. (2022) [62] noted that the best-characterized mechanism of antioxidant properties of flavonoids is related to their ability to interact with ROS by scavenging or reducing them. Flavonoids can display numerous actions depending on their structure—antioxidant, anti-inflammatory, anti-allergic, antiplatelet aggregation, anti-atherogenic, anti-angiogenic, anti-allergic, blood vessel-dilating, lipid-normalizing, antimicrobial, and/or anti-hyperglycemic [63,64]. Positive correlations among TFC, total phenolic content (TPC), and antioxidant activity were determined in a previous study [38]. In addition, a study revealed that the 541 and 463 half-sib families of Norway spruce contained the highest concentrations of organic acids and TPC. The extracts of the needles of these families also exhibited high DPPH and ABTS antioxidant properties. Moreover, a previously conducted pilot study performed by Sirgedaitė-Šėžienė et al. (2021) [37] indicated that the TPC production in the needles of the 477, 463, and 541 half-sib families was strongly stimulated by the short-term (1 min) CP effect. A substantial increase in the concentration of biologically active compounds and antiradical activity in the leaves of a medical plant—*Echinacea purpurea* (L.)—was determined in the study of Mildažienė et al. (2017) [65]. The authors indicated that seed treatments also strongly increased the radical scavenging activity (by up to 114%, according to DPPH assays) in the leaf extracts of the medical plant purple coneflower. Depending on the chemical reactions involved, antiradical assays could be divided into two categories of hydrogen atom transfer (HAT) reaction-based assays, and single electron transfer (ET) reaction-based assays. DPPH is an ET-based method with the HAT mechanism only being a marginal reaction pathway in the assay [66]. This method is mainly based on the prediction that antiradical activity is equal to its electron donating capacity or so-called reducing power. The results of the study by Shahidi and Zhong (2015) [67] show that the detection of antiradical activity in different kinds of foods and beverages using the DPPH antiradical assay can be considered a validated method for certain types of foods. The ABTS assay is usually classified as an ET-based method, and the HAT mechanism also applies. The ABTS assay could be used for the measurement of the total antiradical activity in pure substances, body fluids, and plant materials. Valu et al. (2021) [68] found that ABTS can be solubilized in both hydrophilic and lipophilic media and is not affected by the ionic strength of the medium; however, the DPPH assay is more suitable for fats and oils [69]. The analysis of the variance components of the treatment effect showed that the strongest effect was for the antioxidative activity detected by the ABTS test. The family effect was strong for the antioxidative activity determined by the DPPH binding test and was 4–10-fold depending on the trait. The results of our findings indicate a statistically significant effect on antioxidant activity with both the DPPH and ABTS radical scavenging assays, as well as on the flavonoid content after short-term treatment with CP in extracts of 541 *P. abies* half-sib family needles. The higher antioxidant activity using other methods (FRAP and ORAC tests) was determined in the freeze-dried and frozen material of *P. abies* needles [6]. Jyske et al. found that the vitamins, minerals, and antioxidant activity of freshly frozen Norway spruce sprouts and needles were well retained during the two-year storage period (–20 °C). These results indicate that freshly frozen Norway spruce sprouts could be stored for long periods of time, and thus, entrepreneurs could balance the annual harvest, storage, and the quantity of the final product manufacturing. Sprouts and new developing shoots of *P. abies* are rich in vitamin C and health-promoting minerals [6], and therefore, the dietary intake of these compounds of the sprouts and needles of Norway spruce may have multiple health benefits [15]. 

Organic acids intervene in the pathways of degradation of carbohydrates, amino acids, and fats [70], and may exert a significant impact on human metabolism [71]. For instance, acetic and benzoic acids have exhibited antimicrobial activity [72,73], and citric and succinic acids may be ingredients in the cosmetic safety industry [74,75]. In addition, malic, ascorbic, succinic, citric, and folic acids might be useful for various treatments: malic acid for kidney stones [76], ascorbic acid for providing antioxidant protection against tumor cells [77], and folic acid can interact with NO metabolism and improve digestion [78]. Moreover, some organic acids can be used for the improvement of food taste, and they can extend the expiry dates of various perishable food products. Specific organic acids can also be used to control microbial contamination and the dissemination of foodborne pathogens in both preharvest and postharvest food production [79]. 

Plants are the main source of vitamins needed to maintain the balance of human health. Innovative eco-friendly tools and technologies to increase the production of vitamins, health-promoting organic acids, and micro- and macronutrients in plants are under constant development. 

## 5. Conclusions

As shown in our study, physical stressors such as cold plasma can be used as an alternative technology to induce the positive selective modulation of numerous biochemical processes in growing seedlings and thus stimulate the production of antioxidants and the biosynthesis of health-promoting organic acids. In this study, we established that bioactive compounds (flavonoids) and antioxidant activity were higher in the 541 half-sib family of Norway spruce needles after seed treatment with CP1 compared to the other half-sib families. In addition, it was found that the accumulation of five organic acids in the 541 half-sib family of Norway spruce also was related to short-term (1 min) treatment with CP. Interestingly, the statistically significant positive CP2 effects were determined based on the content of ascorbic acid in many of the treated Norway spruce half-sib families. The continuous assessment of cold plasma is highly needed and prospective since the accumulation of bioactive compounds in trees depends on both the treatment time and genetic background of trees. Alternative eco-friendly technologies, such as cold plasma, could be used to stimulate the synthesis of bioactive chemical compounds in woody plant tissues, which are increasingly used in the medical and dietary supplement industries.

## Figures and Tables

**Table 1:** Total number (n) of Norway spruce samples.

Flavonoids and antioxidant activity	7 half-sib families × 3 different treatment	3 biological replicates	n = 63
Organic acids	7 half-sib families × 3 different treatment	1 biological replicates	n = 21

**Table 2:** Contribution of the traits (%) to the 1st and 2nd components.

Trait	1st Component	2nd Component
Flavonoids	14.63	16.92
DPPH	8.48	14.99
ABTS	14.00	19.09
Oxalic	0.07	0.31
Malic	21.90	6.19
Ascorbic	4.67	16.31
Folic	9.38	14.30
Citric	0.01	7.19
Succinic	26.86	4.70

**Table 3:** Pairwise comparison between the treated seeds of Norway spruce half-sib families and the control. Sample size for organic acids: n = 21; the rest: n = 63.

Trait	Compared Variants	*t* Value	*p* Value
Flavonoids	CP1 vs. control	0.41	0.6802
	CP2 vs. control	−1.25	0.2150
	CP1 vs. CP2	1.86	0.0654
DPPH	CP1 vs. control	4.55	<0.0001
	CP2 vs. control	−0.85	0.3959
	CP1 vs. CP2	4.58	<0.0001
ABTS	CP1 vs. control	−0.28	0.7824
	CP2 vs. control	−4.28	<0.0001
	CP1 vs. CP2	4.27	<0.0001
Oxalic	CP1 vs. control	2.17	0.0364
	CP2 vs. control	0.87	0.3923
	CP1 vs. CP2	0.84	0.4043
Malic	CP1 vs. control	1.42	0.1644
	CP2 vs. control	−0.38	0.7025
	CP1 vs. CP2	1.68	0.1037
Ascorbic	CP1 vs. control	6.37	<0.0001
	CP2 vs. control	3.34	0.0026
	CP1 vs. CP2	1.82	0.0766
Folic	CP1 vs. control	0.71	0.4840
	CP2 vs. control	0.81	0.4234
	CP1 vs. CP2	−0.04	0.9710
Citric	CP1 vs. control	1.59	0.1200
	CP2 vs. control	0.46	0.6476
	CP1 vs. CP2	1.13	0.2636
Succinic	CP1 vs. control	3.09	0.0052
	CP2 vs. control	1.08	0.2873
	CP1 vs. CP2	2.69	0.0131

## Data Availability

Maindata are provided in an article. Detailed calculations are not provided since extremely big amount of the data. The whole package of the data is available upon request.

## Abbreviation

ABTS	2:2’-azino-bis (3-ethylbenzothiazoline-6-sulfonic acid)
CP	cold plasma
DBD	dielectric barrier discharge
DPPH	2,2-diphenyl-1-picryl-hydrazyl-hydrate
NO	nitric oxide
TFC	total flavonoid content
